# Can Aquatic Plant Turions Serve as a Source of Arabinogalactans? Immunohistochemical Detection of AGPs in Turion Cells

**DOI:** 10.3390/molecules30244689

**Published:** 2025-12-07

**Authors:** Bartosz J. Płachno, Lubomír Adamec, Marcin Feldo, Piotr Stolarczyk, Małgorzata Kapusta

**Affiliations:** 1 Department of Plant Cytology and Embryology, Institute of Botany, Faculty of Biology, Jagiellonian University, 9 Gronostajowa St., 30-387 Kraków, Poland; 2 Institute of Botany of the Czech Academy of Sciences, Dukelská 135, CZ-379 01 Třeboň, Czech Republic; 3 Department of Vascular Surgery and Angiology, Medical University of Lublin, 16 Staszica St., 20-081 Lublin, Poland; 4 Department of Botany, Physiology and Plant Protection, Faculty of Biotechnology and Horticulture, University of Agriculture in Kraków, 29 Listopada 54 Ave., 31-425 Kraków, Poland; 5 Bioimaging Laboratory, Faculty of Biology, University of Gdańsk, 59 Wita Stwosza St., 80-308 Gdańsk, Poland

**Keywords:** *Aldrovanda*, arabinogalactan proteins, aquatic plants, *Caldesia*, carnivorous plants, cell wall, turions, *Utricularia*, waterwheel plant

## Abstract

Turions (overwintering buds) as modified shoot apices constitute specialized vegetative structures that enable many aquatic vascular plants to withstand adverse environmental conditions such as low temperature, desiccation, or limited light availability. Turions serve as major storage sites for organic reserves, including sugars, proteins, fatty acids, and polyamines. Owing to their high content of energy-rich and nutritionally valuable compounds, turions represent a potential renewable resource for applications in biofuel production, animal feed, and the food industry. We investigated whether arabinogalactan proteins (AGPs) occur in aquatic plant turions and localized these compounds within specific tissues or cell types. This work was designed to evaluate whether stress-resistant storage organs may constitute a practical reservoir of AGPs. Considering the central role of AGPs in plant responses to abiotic stress, we hypothesized that turions, which routinely encounter cold, anoxia, and intermittent dehydration, would exhibit particularly high AGP accumulation. Mature turions of aquatic species (*Aldrovanda vesiculosa*, *Utricularia australis*, *U. intermedia*, and *Caldesia parnassifolia*) were used. Immunofluorescent labeling with AGP-specific antibodies (JIM8, JIM13, JIM14, LM2, MAC207) and confocal laser scanning microscopy were employed. In *Aldrovanda vesiculosa* and *Caldesia parnassifolia*, AGP epitopes were abundantly presented in cytoplasmic compartments. AGP epitopes occurred in secretory structures in turions of all examined species (trichomes of *Aldrovanda* and *Utricularia*, secretory ducts of *Caldesia*). In analyzing turions of four different species, we identified *Aldrovanda vesiculosa* turions as the most promising potential source of AGPs, also noting their high reserve potential for use in animal feed or the food industry.

## 1. Introduction

Turions (dormant buds) are morphologically distinct, vegetative overwintering, or stress-resistant organs produced by many aquatic vascular plants in response to unfavorable environmental conditions, such as low temperature, reduced irradiance, short photoperiod, and desiccation [1,2,3,4,5,6]. However, turions function not only as dormant organs but also as vegetative dispersal propagules [1], which can be transported by water currents or, analogously to seeds, by animals, attached to fur or feathers, or even following passage through the digestive tract [7,8]. Although the term turion has sometimes been applied to various plant structures, Adamec [5] emphasized that it should refer exclusively to modified, above-ground, detachable, green aquatic shoots capable of photosynthesis. Turions typically develop through condensation of shoot apices with abbreviated internodes and modified leaves, scales, or phylloclades, and they always detach from the maternal plant sooner or later [1,5].

Turion formation has evolved independently across multiple lineages of aquatic angiosperms. According to Adamec [5], at least 14 genera spanning nine families, including Ceratophyllaceae, Potamogetonaceae, Araceae, Cabombaceae, Hydrocharitaceae, Lentibulariaceae, and Droseraceae, possess this trait. Therefore, turions represent a case of convergent functional adaptation, although they differ considerably in ecological strategy. Most turions overwinter submerged in dark, hypoxic, or anoxic sediments and resume growth when favorable environmental conditions return [5,9].

Turions also serve as storage organs, accumulating diverse organic compounds—most prominently carbohydrates such as starch and soluble sugars [10,11,12,13,14]. Starch and free sugars have been identified as the principal reserve substances in mature turions of 21 aquatic plant species [14]. Starch functions as the main energy reserve for overwintering metabolism: during dormancy, respiration continues at a low rate sustained by gradual starch degradation [9,15,16]. It also influences turion buoyancy, as starch density affects sinking and floating behavior [17]. Partial hydrolysis of starch into soluble sugars (sucrose, glucose, fructose) provides cryoprotection, increasing osmotic potential, lowering the freezing point, and enhancing dehydration tolerance. When temperatures rise, starch is rapidly mobilized to support early growth before photosynthesis resumes [5]. Owing to their high starch content, turions have even been proposed as a novel biomass source for biofuel production [18].

Turions may also contain polyamines [19] and lipids, typically in the form of lipid bodies [13]. Lipid metabolism in turion cells is particularly notable. Strzemski et al. [20] demonstrated that turions of *Utricularia* species contain approximately 50% fatty acids, suggesting potential use as a source of oil rich in unsaturated fatty acids beneficial to humans. Furthermore, turions contain storage proteins, often in the form of protein storage vacuoles (PSVs), as observed in *Utricularia* turions [13]. Proteins have also been reported as crystalline inclusions within the nuclei of turion cells [13,21,22].

The high content of reserve compounds makes turions a valuable food resource for aquatic birds [5]. In summary, due to their significant content of proteins, starch, and fatty acids, turions may have potential applications as animal feed or in the food industry. However, current knowledge of the diversity and composition of organic storage substances in turions remains limited and warrants further investigation. Therefore, we aimed to examine whether arabinogalactan proteins (AGPs) are present in turions.

Arabinogalactan proteins (AGPs) are glycoproteins featuring a PAST-rich (proline, alanine, serine, threonine) protein domain with repetitive dipeptides, covalently linked type II arabinogalactans, an N-terminal secretion signal, a C-terminal GPI anchor for membrane attachment, and the ability to bind the β-Yariv reagent, which recognizes the β-1,3-galactan backbone of their arabinogalactan chains. They are localized mainly at the plant cell membrane and within the extracellular matrix, including the cell wall, intercellular spaces, and soluble secretions [23,24,25,26].

Functionally, AGPs play critical roles in plant growth, development, and cell communication. They are involved in cell expansion, embryogenesis, xylem differentiation, pollen tube guidance, somatic embryogenesis, and responses to abiotic and biotic stress [27,28,29,30,31]. AGPs are also essential for sexual reproduction, participating in both male and female gametophyte development [32,33,34,35,36,37]. Moreover, AGPs have been implicated in fruit ripening and post-harvest physiology, influencing texture and shelf life [38,39,40,41]. Beyond plant physiology, AGPs attract an attention for their nutritional and pharmaceutical potential; for example, wheat AGPs have demonstrated prebiotic activity [42]. In the case of animals and humans, arabinogalactan proteins have been reported to exhibit a range of biological activities, including anti-diabetic, immunomodulatory, antioxidant, and antitumor properties, e.g., [43,44,45,46,47,48].

The objective of the present study was to determine whether AGPs occur in mature aquatic plant turions and to localize these compounds within specific tissues or cell types. In our study, we wanted to identify storage-resistant organs as a potential source of AGPs that could be used in practical applications. Since AGPs play an important role in plant responses to stress, we propose the hypothesis that organs such as turions (which are exposed to stress related to cold, anaerobic conditions, or temporary drying) will be rich in AGPs. Immunofluorescent labelling with AGP-specific antibodies and confocal laser scanning microscopy (CLSM) were employed to visualize their distribution at the cellular level. As AGPs play essential roles for plant growth and development and as turions represent storage organs with high reserve potential, we also consider whether turions might be a source of AGPs or serve as an animal feed or for the food industry. Of the four species tested, *Aldrovanda vesiculosa* turions appeared to be the most promising potential source of AGPs.

## 2. Results

### 2.1. Characterization of Turions in Aquatic Plants

**Figure 1:**
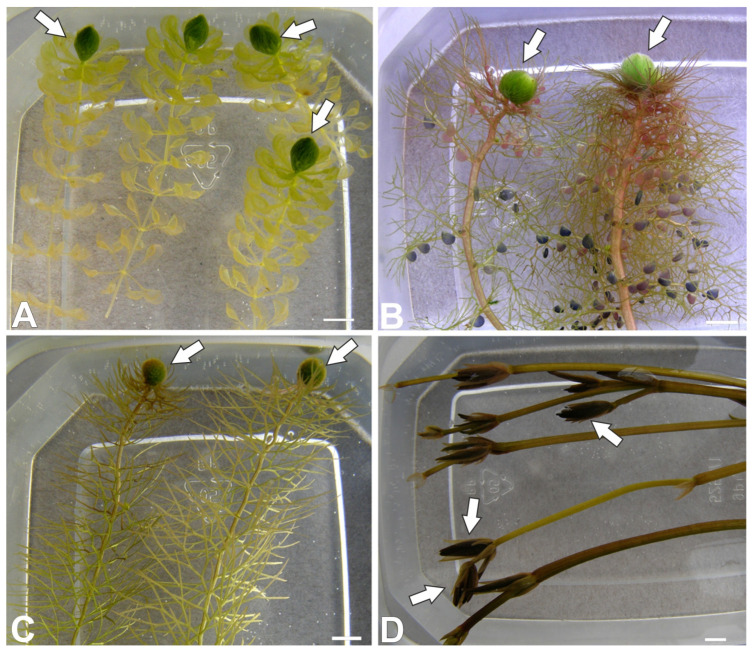
Examined plant species forming turions. (**A**) *Aldrovanda vesiculosa* plants with turions (arrows). (**B**) Two *Utricularia australis* shoots with turions (arrows). (**C**) Two *Utricularia intermedia* shoots with turions (arrows). (**D**) *Caldesia parnassifolia* shoots with turions (arrows). All bars 5 mm.

**Figure 2:**
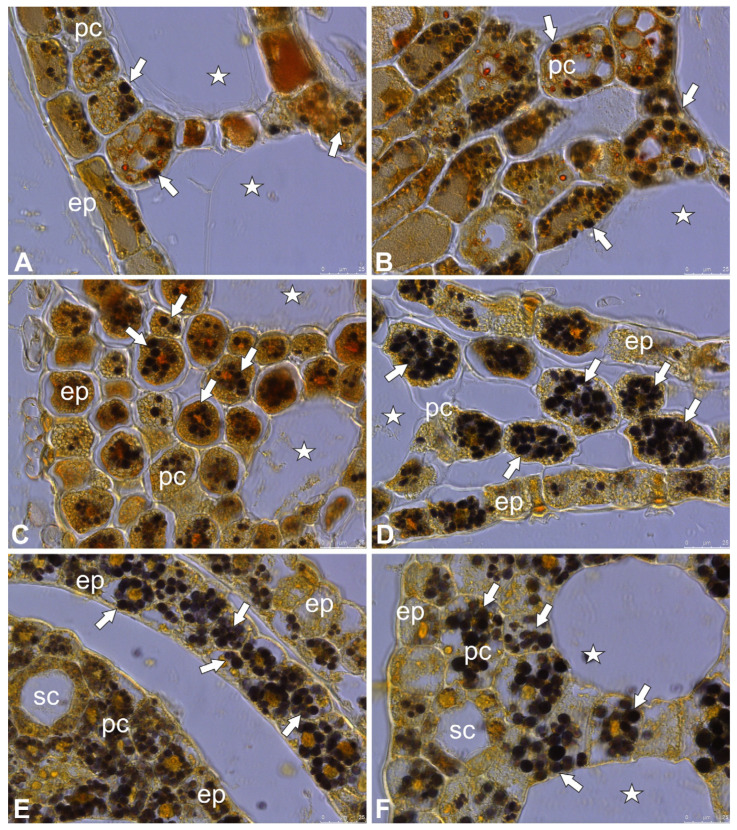
Bright-field images of turions, Lugol’s iodine staining. (**A**,**B**) Cross section of leaf of *Aldrovanda vesiculosa*; epidermal cells (ep), parenchyma cells (pc), starch grains (arrows), air duct (star). (**C**) Cross section of phylloclade of *Utricularia australis*; epidermal cell (ep), parenchyma cells (pc), starch grains (arrows), air duct (star). (**D**) Cross section of phylloclade of *Utricularia intermedia*; epidermal cell (ep), parenchyma cells (pc), starch grains (arrows), air duct (star). (**E**,**F**) Cross section of leaves of *Caldesia parnassifolia*, epidermal cells (ep), parenchyma cells (pc), starch grains (arrows), secretory canal (sc), air duct (star).

Turions (Figure 1A–D) were formed in apical parts of the vegetative shoots and the on flower stem in the case of *Caldesia parnassifolia*. *Aldrovanda vesiculosa* turions consist of a shortened shoot with leaves with immature traps. *Utricularia* turions consist of a shortened shoot with phylloclades with immature traps. *Caldesia parnassifolia* turions consist of a shortened shoot with scaly leaves. Lugol’s iodine staining showed that the cells of turions in all species studied contained amyloplasts (Figure 2A–F) (compared with the results in [13]).

### 2.2. AGP Detection in Aldrovanda vesiculosa

**Figure 3:**
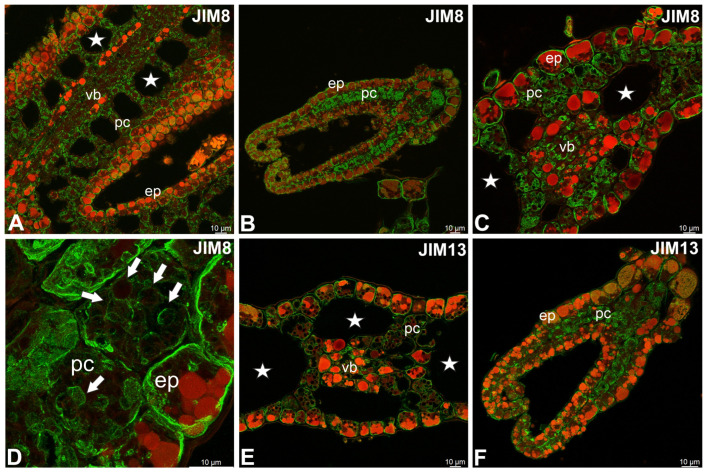
Distribution of arabinogalactan proteins (JIM8, JIM13) detected in the turion of *Aldrovanda vesiculosa* (intense green color—antibody signal; red-brown color—autofluorescence); epidermal cells (ep), parenchyma cells (pc), vascular bundle (vb), air duct (star). (**A**) Occurrence of JIM8 epitopes in stem. (**B**) Occurrence of JIM8 epitopes in trap; note intense signal in the parenchyma cells. (**C**) Occurrence of JIM8 epitopes in parenchyma cells and vascular bundle cells. (**D**) Occurrence of JIM8 epitopes in intracellular compartments (white arrows) of parenchyma and epidermal cells. (**E**) Occurrence of JIM13 epitopes in leaf; note that signal occurred in epidermal and parenchyma cells and vascular bundle cells. (**F**) Occurrence of JIM13 epitopes in trap; note intense signal in the parenchyma cells.

**Figure 4:**
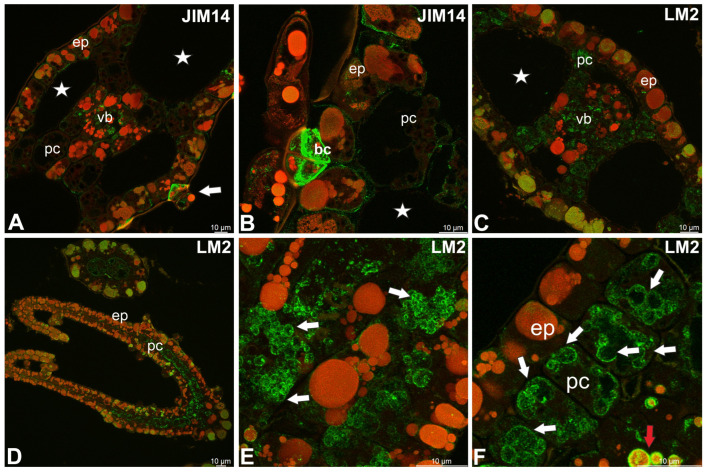
Distribution of arabinogalactan proteins (JIM14, LM2) detected in the turion of *Aldrovanda vesiculosa* (intense green color—antibody signal; red-brown color—autofluorescence); epidermal cells (ep), parenchyma cells (pc), vascular bundle (vb), air duct (star). (**A**) Occurrence of JIM14 epitopes in leaf, trichome (white arrow). (**B**) Occurrence of JIM14 epitopes in glandular trichome; note intensive signal in basal cells of trichome (bc). (**C**) Occurrence of LM2 epitopes in leaf. (**D**) Occurrence of LM2 epitopes in trap. (**E**,**F**) LM2 epitopes in intracellular compartments (white arrows) including vacuoles (red arrow).

JIM8 epitopes were recorded in epidermal cells, glands, parenchyma cells, and vascular tissues (Figure 3A–C). JIM8 epitopes were associated with cell walls and intracellular compartments, such as plastids (amyloplasts) and some vacuoles (Figure 3D). Similar labeling was observed in case JIM13 epitopes (Figure 3E,F). JIM14 epitopes were present in the wall/plasma membrane in both epidermal and parenchymal cells (Figure 4A,B). JIM14 epitopes were especially abundant in basal cells of glandular trichomes (Figure 4B). These epitopes also occurred in vascular tissue (Figure 4A). The LM2 epitopes also occurred in vascular tissue (Figure 4C) and in the cytoplasmic compartments of various cells (such as plastids and some vacuoles) (Figure 4D–F). MAC207 epitopes were not found.

### 2.3. AGP Detection in Utricularia australis and U. intermedia

**Figure 5:**
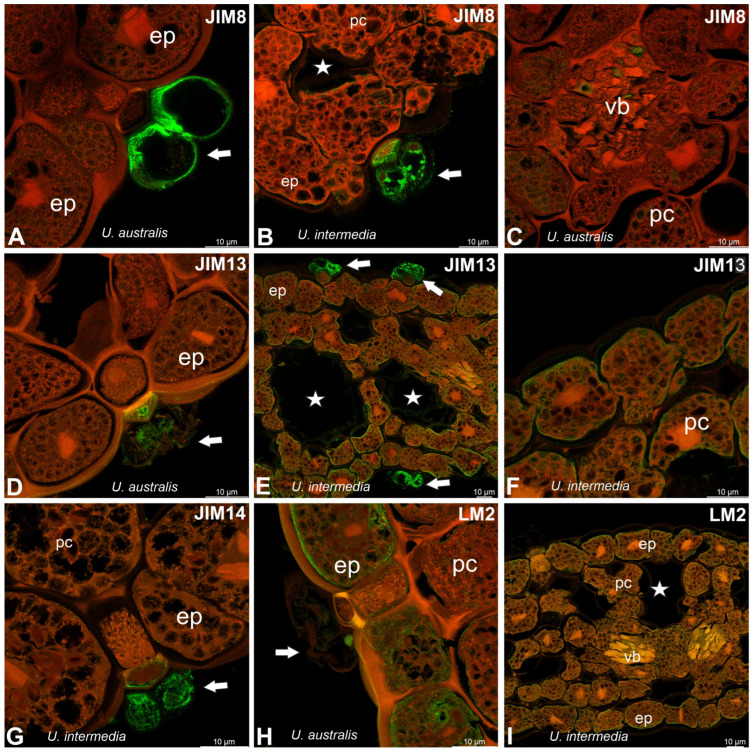
Distribution of arabinogalactan proteins (JIM8, JIM13, JIM14, LM2) detected in the turion of *Utricularia* (intense green color—antibody signal; red-brown color—autofluorescence); epidermal cells (ep), parenchyma cells (pc), vascular bundle (vb), air duct (star), glandular trichome (arrow). (**A**) Occurrence of JIM8 epitopes in glandular trichomes of *Utricularia australis*. (**B**) Occurrence of JIM8 epitopes in glandular trichomes of *Utricularia intermedia*. (**C**) Occurrence of JIM8 epitopes in vascular bundle of *Utricularia australis*. (**D**) Occurrence of JIM13 epitopes in glandular trichomes of *Utricularia australis*. (**E**) Occurrence of JIM13 epitopes in phylloclade of *Utricularia intermedia*; note positive signal in glandular trichomes. (**F**) Positive signal in parenchyma cells of *Utricularia intermedia*. (**G**) Occurrence of JIM14 epitopes in glandular trichome of *Utricularia intermedia*. (**H**) Occurrence of LM2 epitopes in epidermal cells of *Utricularia australis*. (**I**) Occurrence of LM2 epitopes in phylloclade of *Utricularia intermedia*.

In both species, JIM8 epitopes were recorded in glandular trichomes (Figure 5A–C). In *U. australis*, JIM8 epitopes were recorded in cells of vascular tissues (Figure 5C). In both species, JIM13 epitopes were recorded in glandular trichomes (Figure 5D,E). In *Utricularia intermedia* cells, which were plasmolyzed, the JIM13 epitopes were presented in the plasma membrane (Figure 5F). The JIM14 epitopes occurred especially in glandular trichomes cells and as small dots on plasma membranes of epidermal cells (Figure 5E).

LM2 epitopes were present in the plasma membrane (Figure 5H,I). MAC207 epitopes were not found.

### 2.4. AGP Detection in Caldesia parnassifolia

**Figure 6:**
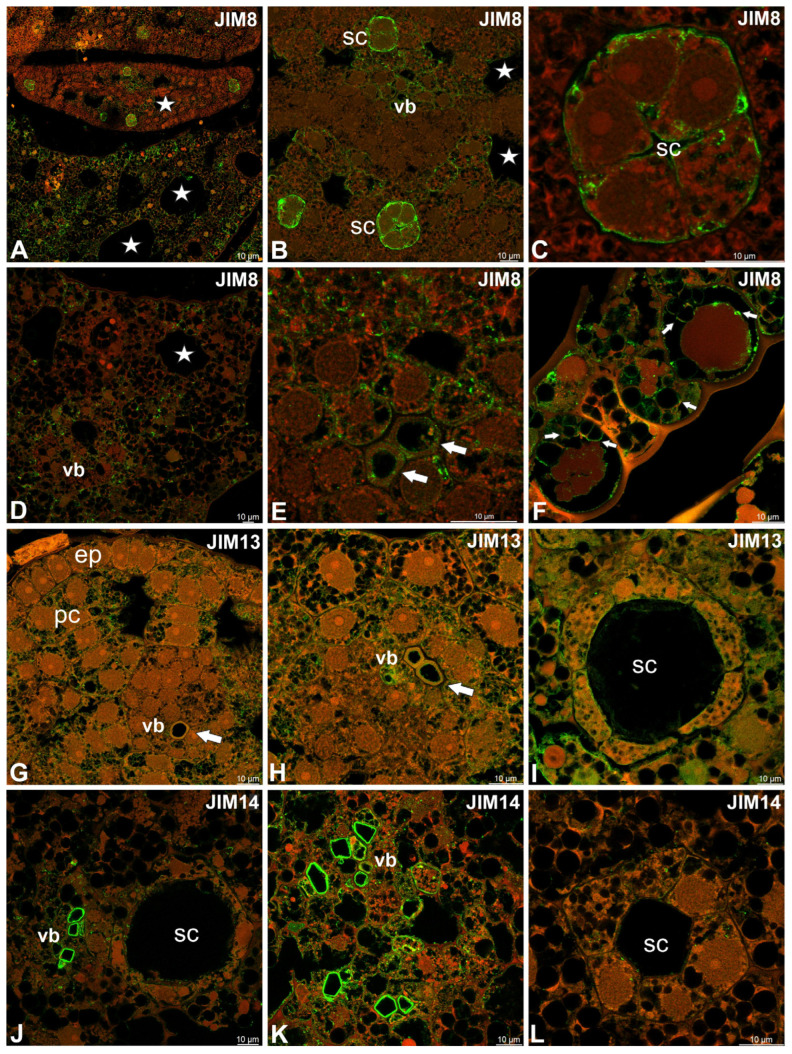
Distribution of arabinogalactan proteins (JIM8, JIM13, JIM14) detected in the turion of *Caldesia parnassifolia* (intense green color—antibody signal; red-brown color—autofluorescence); epidermal cells (ep), parenchyma cells (pc), secretory canal (sc), air duct (star). (**A**) Cross sections of turion leaves, showing occurrence of JIM8 epitopes. (**B**,**C**) Occurrence of JIM8 epitopes in young secretory ducts. (**D**,**E**) Occurrence of JIM8 epitopes in vascular bundles (vb), with xylem tracheary elements (white arrows). (**F**) JIM8 epitopes in the cytoplasmic compartments (white arrows). (**G**) JIM13 epitopes in cells of turion leaves. (**H**) JIM13 epitopes in cells of vascular bundle (white arrow) and parenchyma cells. (**I**) JIM13 epitopes in secretory ducts. (**J**,**K**) JIM14 epitopes in vascular bundle; note intensive signal in some cells. (**L**) JIM14 epitopes in secretory ducts.

The AGP epitopes that are recognized by JIM8 were present in epidermal and parenchyma cells (Figure 6A,B). A very intensive signal of JIM8 was in the cell walls of secretory duct cells (Figure 6B,C). JIM8 epitopes were recorded in cells of vascular tissues and in xylem elements (Figure 6D,E). JIM8 epitopes were abundantly present in the cytoplasmic compartments (Figure 6F).

**Figure 7:**
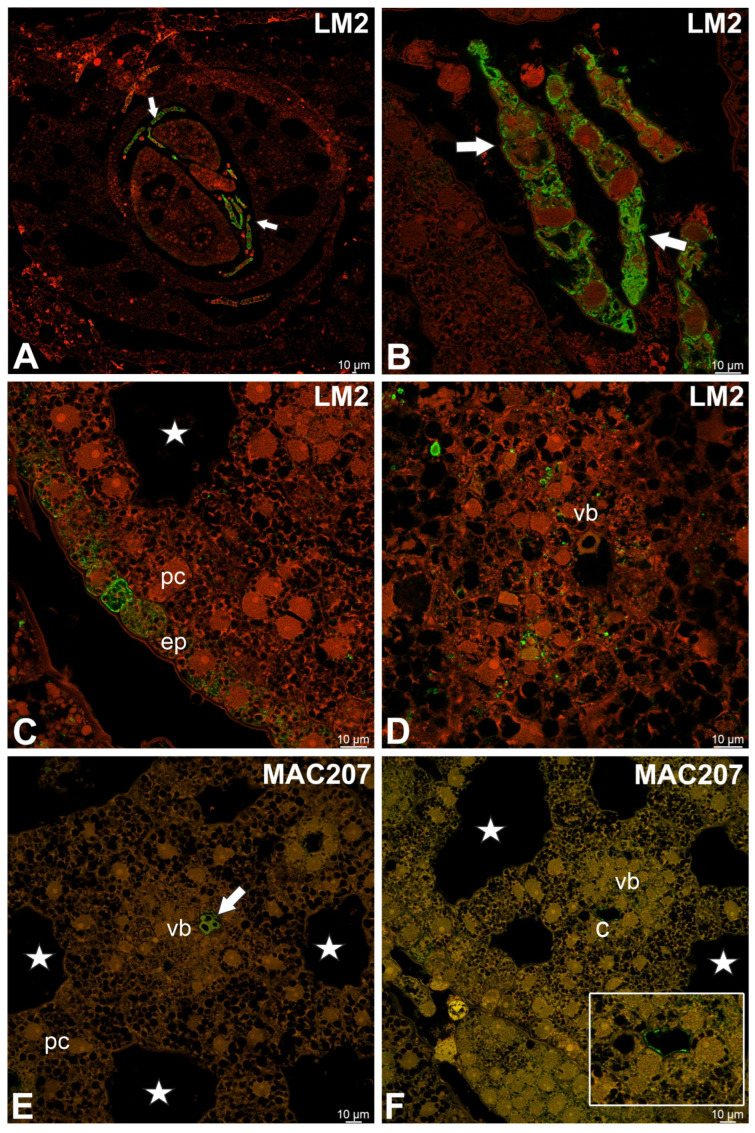
Distribution of arabinogalactan proteins (LM2, MAC207) detected in the turion of *Caldesia parnassifolia* (intense green color—antibody signal; red-brown color—autofluorescence); epidermal cells (ep), parenchyma cells (pc), vascular bundle (vb), air duct (star). (**A**) Cross sections of turion leaves; note strong antibody signal (LM2) in epidermal outgrowths (white arrows). (**B**) Epidermal outgrowths (white arrows); note AGPs (LM2) detected in the cytoplasmic compartments. (**C**) AGPs (LM2) detected in epidermal cells. (**D**) AGPs detected in the cytoplasmic compartments in various cells. (**E**) AGPs (MAC207) detected in tracheary elements of xylem (white arrow). (**F**) AGPs (MAC207) detected inside canal (c); see magnified in the panel.

JIM13 epitopes were present in parenchyma cells in the cytoplasmic compartments (Figure 6G) and in some cells in vascular bundles (Figure 6H). JIM13 epitopes also occurred in the secretory duct cells (Figure 6I). JIM14 epitopes were mainly present in vascular bundles (Figure 6J,K). However, the JIM14 epitopes also occurred in the cytoplasmic compartments of various cells (signals seen as dots), including secretory duct cells (Figure 6L). LM2 epitopes occurred abundantly in epidermal outgrowths (Figure 7A,B). LM2 epitopes also occurred in epidermal cells (Figure 7C) and in the cytoplasmic compartments of various cells (signals seen as dots) (Figure 7D). MAC207 epitopes were in xylem elements (Figure 7E) and in canals (Figure 7F).

### 2.5. Summary

The results of immunocytochemical patterns across species is given in Table 1.

## 3. Discussion

Our study demonstrated that arabinogalactan proteins (AGPs) in aquatic plant turions show a complex pattern of tissue and subcellular localization that is consistent with, yet extends beyond, previous observations in a range of plant taxa. AGP epitopes were recorded not only in cell walls, where their presence is expected due to their association with the plasma membrane or secretion into the apoplast [28,49], but also in multiple intracellular compartments. This intracellular localization aligns with the biosynthetic route of AGPs, whose glycans are assembled in the Golgi apparatus and transported in secretory vesicles [50,51,52]. Comparable patterns were previously observed in integument cells of *Taraxacum*, where JIM13-reactive AGPs occurred within the endomembrane system [53], and in *Arabidopsis thaliana* embryos and explants, in which LM2- and JIM4-reactive epitopes were detected inside storage-rich cells [54,55]. Similar to those systems, turion cells, also rich in storage materials [13], contained AGP epitopes in plastids, including amyloplasts, and in protein storage vacuoles.

The association of AGPs with vacuoles appears to be a recurring theme across taxa. Earlier studies documented AGP epitopes in vacuoles with calcium oxalate crystals in *Fragaria* × *ananassa* [56,57] and in parenchyma cells of *Pilosella officinarum* ovules [58], suggesting potential roles in crystal formation or stabilization. In our material, JIM8 epitopes were likewise found in vacuoles of *Aldrovanda vesiculosa* and *Caldesia parnassifolia*. Reports of *Trithuria submersa*, where JIM8, LM2, and JIM13 epitopes occurred on the outer surfaces or even within starch grains of multiple tissues [59], further support the notion that AGPs participate in organizing storage-related compartments. Although this intracellular presence may reflect secretory or trafficking pathways, ultrastructural analyses would be essential to confirm whether AGPs are embedded in the starch–grain matrix or only associated with surrounding membranes.

A substantial proportion of AGP localization in our study concerned secretory structures, particularly glandular trichomes. Turions of both *Utricularia* species contained AGPs in these specialized cells, consistent with earlier findings from stolons and leaf-like shoots of *Utricularia neottioides* [60], as well as from quadrifid trichomes and external glands of *U. dichotoma* traps [61,62]. In *Aldrovanda vesiculosa* turions, AGP epitopes detected with the JIM14 antibody were present in trichomes homologous to bifid trichomes found in *Aldrovanda* traps, where AGPs had already been documented [63]. Integrating our observations with studies on carnivorous genera, such as *Drosophyllum* [64], *Dionaea* [65,66,67], and *Drosera* [55], suggests that AGPs may play consistent functional roles in both mucilage-producing and digestive glands. Their abundance in these structures likely reflects extensive wall remodeling associated with secretion, including wall ingrowth deposition and secondary wall development. This interpretation accords with demonstrations that AGPs are associated with the endoplasmic reticulum and Golgi apparatus of actively secreting cells [55] and that they coordinate cell wall ingrowth formation in transfer cells [68].

AGP epitopes were also detected in internal secretory ducts of *Caldesia parnassifolia* turions. Such ducts, previously described for this species [68] and typical for the Alismataceae family [69,70], produce exudates containing lipids, alkaloids, proteins, and polysaccharides, including mucilage. Comparable AGP localizations have been reported in other internal secretory systems, including LM2-reactive epitopes in resin–duct sheath cells of *Pinus pinaster* [71], JIM13 epitopes in mucilage cells of *Araucaria angustifolia* [72], and various AGP epitopes in mucilage cells of *Taraxacum officinale* [60]. AGPs also occur in seed mucilage of *Arabidopsis thaliana* [73,74], further underscoring their widespread involvement in secretory processes.

Our data also show that AGPs occur in the vascular tissues of turions, which aligns with reports from diverse angiosperm and gymnosperm species. In *Populus trichocarpa*, LM2 epitopes were present in all tissues of secondary roots, including cells with primary and secondary lignified walls, whereas in primary roots, AGPs were absent from xylem [75]. Other species exhibit different patterns: AGPs were recorded in protoxylem of *Ceratopteris richardii*, *Picea sitchensis,* and *P. trichocarpa*, while in *Zea mays* immunolabeling was strongest in xylem parenchyma surrounding metaxylem vessels [76]. By contrast, herbaceous *Arabidopsis thaliana* lacked detectable AGPs in xylem [76]. Additional studies showed LM2 epitopes in metaxylem and JIM13, JIM14, and MAC207 epitopes in metaphloem sieve elements and companion cells [77]. In *Pinus pinaster*, LM2-reactive epitopes accumulated in the phloem [71]. Collectively, these findings suggest that AGPs participate in vascular differentiation, autophagy, and wall modification processes [76,78], and our observations extend these roles to aquatic overwintering organs.

Altogether, the results indicate that turions of aquatic plants, particularly those of *Aldrovanda vesiculosa*, harbor AGPs across a wide array of tissues and intracellular compartments. This broad distribution has implications not only for developmental biology but also for the search for new sources of arabinogalactans. At present, the principal commercially exploited sources of arabinogalactans include gum exudates of *Acacia senegal* and *A. seyal* [79,80], gums of Rosaceae, such as *Prunus* spp. and *Amygdalus scoparia* [81], and conifer wood, particularly *Larix* species, which produce well-characterized, water-soluble arabinogalactans with beta-(1→3) backbones and beta-(1→6) side chains [82]. While herbaceous plants and their organs can also serve as sources [43], aquatic turions have not been explored in this context. Our findings suggest that *Aldrovanda* turions, owing to their rapid multiplication in in vitro culture [83] and the species’ known production of diverse secondary metabolites [84], may offer a promising alternative resource. However, our study only demonstrates the presence of specific AGP epitopes; subsequent research must address the quantities, extractability, and biochemical properties of turion-derived AGPs.

Future work should therefore include comprehensive extraction and structural analyses, ultrastructural verification of subcellular AGP localization and functional assays evaluating potential applications. Such efforts will help determine whether aquatic turions can be considered a viable, sustainable source of arabinogalactans for biotechnological or industrial purposes, while also contributing to a deeper understanding of AGP biology in aquatic plants.

## 4. Materials and Methods

### 4.1. Plant Material and Sample Collection

The plant material, mature turions of *Aldrovanda vesiculosa* L. (voucher PL 0 HBT 2017.04079, Droseraceae), *Utricularia australis* R.Br. (CZ 0 HBT 2017.04057, *Utricularia intermedia* Hayne (CZ 0 HBT 2017.04058, Lentibulariaceae), and *Caldesia parnassifolia* (L.) Parl. (DE 0 HBT 2017.04000, Alismataceae), were collected from outdoor containers of the collection of aquatic plants in the Institute of Botany CAS at Třeboň. At least four turions of each species were fixed and processed.

### 4.2. Immunolocalization of AGPs

The turions were fixed overnight at 4 °C in 8% (*w*/*v*) paraformaldehyde (PFA, Sigma-Aldrich, Poznan, Poland) with 0.25% (*v*/*v*) glutaraldehyde (GA, Sigma-Aldrich) in a PIPES buffer (Sigma-Aldrich). They were then embedded in Steedman’s wax (PEG distearate and 1-hexadecanol; Sigma-Aldrich) and sectioned into 7 μm sections, which were blocked with 1% BSA (Sigma-Aldrich) in PBS buffer and incubated with the primary antibodies against arabinogalactans (JIM8, JIM13, JIM14, LM2, and MAC207 [85,86,87]) overnight at 4 °C. All of the primary antibodies were purchased from Plant Probes (Leeds, UK) or Kerafast (Newark, CA, USA), and the secondary antibody goat anti-rat conjugated with FITC was purchased from Abcam (Cambridge, UK). The samples were then cover-slipped using a Mowiol mounting medium: a mixture of Mowiol ^®^4-88 (Sigma-Aldrich) and glycerol for fluorescence microscopy (Merck, Warsaw, Poland), with the addition of 2.5% DABCO (Carl Roth GmbH, Karlsruhe, Germany). They were viewed using a Leica STELLARIS 5 WLL confocal microscope with Lightning module deconvolution. Negative controls were created by omitting the primary antibody step, which caused no fluorescence signal in any of the control frames for any stained slides (Figure S1). Confocal z-stacks were acquired using identical imaging parameters for all samples of a given antibody channel. Images were collected in Lightning acquisition mode at a line scan speed of 400–1000 Hz. The FITC channel (antibody labelling) was excited at 494 nm with a detector gain of 2.5 and laser intensity of 9.79%. Turion autofluorescence was recorded using excitation at 540 nm with a detector gain of 2.5 and laser intensity of 3.74%. For each species, four turions were processed and labelled with each antibody, and all biological replicates displayed consistent staining patterns. Each immunolabeling was performed twice. Sections were prepared for light microscopy and stained for starch and protein using Lugol’s iodine stain and later were viewed using a Leica DM6000B microscope (KAWA.SKA Sp. z o.o., Piaseczno, Poland).

## 5. Conclusions

Our study is the first report providing data on AGP occurrence in aquatic plant turions. After analyzing turions of four different species, we identified *Aldrovanda vesiculosa* turions as the most promising potential source of AGPs. Particularly interesting is the finding of AGPs in the cytoplasmic compartments, which may indicate the role of these compounds in the storage function of turions. Since turion cells are filled with starch, protein bodies, and contain secondary metabolites, the presence of AGPs makes them even more interesting organs in terms of their use in the pharmaceutical industry and food production. Our results may serve as a guide for expanding future research directions on turions as a source of AGPs and potential applications, such as quantitative AGP analysis, extraction feasibility, and industrial or biotechnological applications.

## Figures and Tables

**Table 1:** Immunocytochemical patterns across examined species.

	Antibody	JIM8	JIM13	JIM14	LM2	MAC207
Species						
*Aldrovanda* *vesiculosa*		Epidermal cells, glands, parenchyma cells, vascular tissues showed epitopes associated with cell walls and also with intracellular compartments	Epidermal cells, glands, parenchyma cells, vascular tissues showed epitopes associated with cell walls and also with intracellular compartments	Epidermal cells, parenchymal cells, basal cells of glands, vascular tissues showed epitopes associated with the wall/plasma membrane	Parenchymal cells, vascular tissue showed epitopes associated with intracellular compartments	Was not found
*Utricularia australis*		Mainly glandular trichomes and vascular bundle cells	Mainly glandular trichomes	Mainly glandular trichomes	Epidermal cells	Was not found
*Utricularia* *intermedia*		Mainly glandular trichomes	Mainly glandular trichomes, epidermal cells, parenchymal cells	Mainly glandular trichomes, with a weak dotted signal in epidermal cells	Epidermal cells	Was not found
*Caldesia parnassifolia*		Epidermal and parenchyma cells, vascular tissue. Intensive signal in secretory duct cells; epitopes were abundantly present in the cytoplasmic compartments	Parenchyma cells, vascular tissue, secretory duct cells in the cytoplasmic compartments	Mainly present in vascular bundle cells Cell walls, cytoplasmic compartments of various cells	Abundant in epidermal outgrowths, present in epidermal cells and cytoplasmic compartments of various cells	Xylem elements and canals

## Data Availability

Dataset available on request from the authors.

## Supplementary Materials

The following supporting information can be downloaded at https://www.mdpi.com/article/10.3390/molecules30244689/s1. Figure S1. Control reactions of cell wall components after immunolabeling (green color—signal of antibody, red-brown color—autofluorescence), (**A**) Turion of *Aldrovanda vesiculosa*. (**B**) Turion of *Utricularia australis*. (**C**) Turion of *Utricularia intermedia*. (**D**) Turion of *Caldesia parnassifolia*.
