# Supplementary material for: Can Aquatic Plant Turions Serve as a Source of Arabinogalactans? Immunohistochemical Detection of AGPs in Turion Cells
Source: Molecules. 2025 Dec 7;30(24):4689. doi: 10.3390/molecules30244689 (PMC12736068; doi:10.3390/molecules30244689)

Figure S1

Figure S1. Control reactions of cell wall components after immunolabeling (green color – signal of antibody, red-brown color—autofluorescence), (**A**) Turion of *Aldrovanda vesiculosa*. (**B**) Turion of *Utricularia australis*. (**C**) Turion of *Utricularia intermedia*. (**D**) Turion of *Caldesia parnassifolia*.

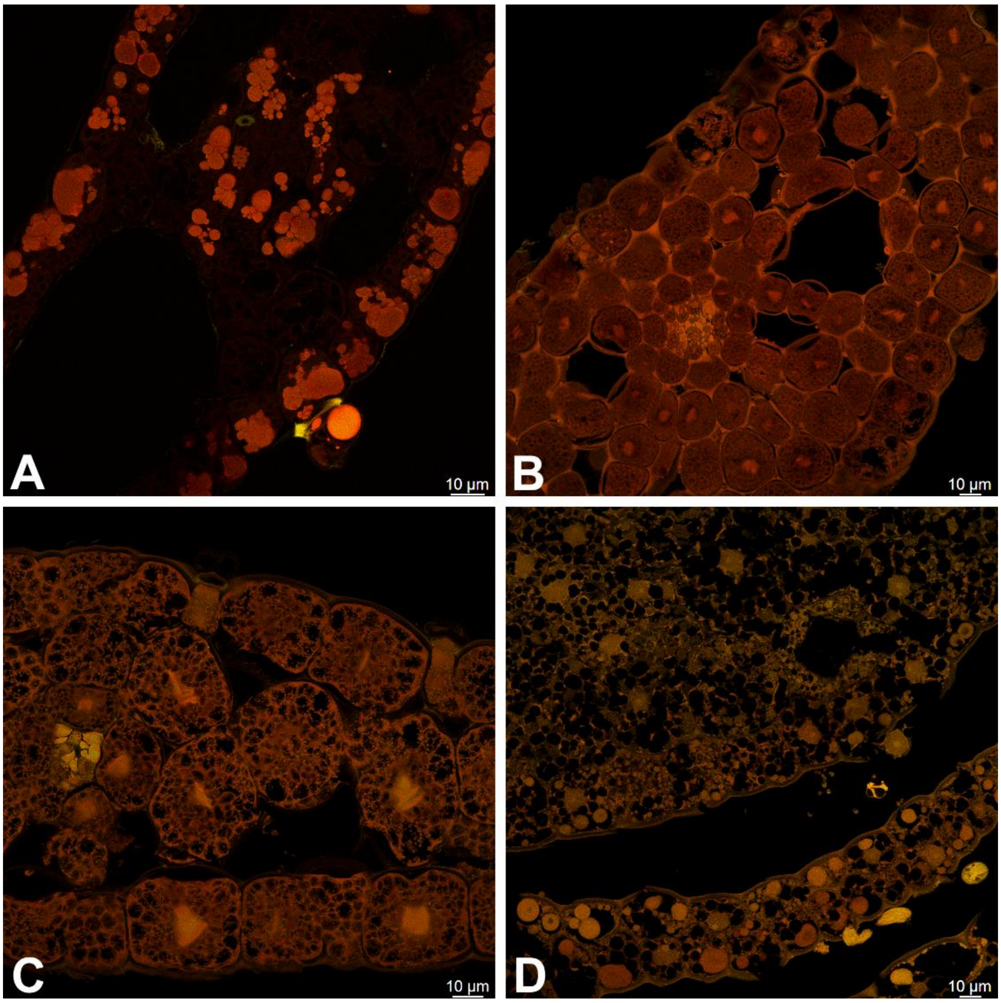

Supplement: Supplementary file 1 [file molecules-30-04689-s001.zip › molecules-3990876-supplementary.pdf]
